# Cu^+^ → Mn^2+^ Energy Transfer in Cu, Mn Coalloyed Cs_3_ZnCl_5_ Colloidal Nanocrystals

**DOI:** 10.1021/acs.chemmater.2c01578

**Published:** 2022-09-20

**Authors:** Ying Liu, Matteo L. Zaffalon, Juliette Zito, Francesca Cova, Fabrizio Moro, Marco Fanciulli, Dongxu Zhu, Stefano Toso, Zhiguo Xia, Ivan Infante, Luca De Trizio, Sergio Brovelli, Liberato Manna

**Affiliations:** † Key Laboratory of Materials Physics of Ministry of Education, School of Physics and Microelectronics, Zhengzhou University, Daxue Road 75, Zhengzhou 450052, China; ‡ Nanochemistry, Istituto Italiano di Tecnologia, via Morego 30, Genova 16163, Italy; § Dipartimento di Scienza dei Materiali, Università degli Studi Milano-Bicocca, via R. Cozzi 55, Milano 20125, Italy; ∥ International Doctoral Program in Science, Università Cattolica del Sacro Cuore, 25121 Brescia, Italy; ⊥ The State Key Laboratory of Luminescent Materials and Devices, Guangdong Provincial Key Laboratory of Fiber Laser Materials and Applied Techniques, School of Physics and Optoelectronics, South China University of Technology, Guangzhou 510641, P. R. China; □ Dipartimento di Chimica e Chimica Industrial, Università degli Studi di Genova, Via Dodecaneso 31, Genova 16146, Italy

## Abstract

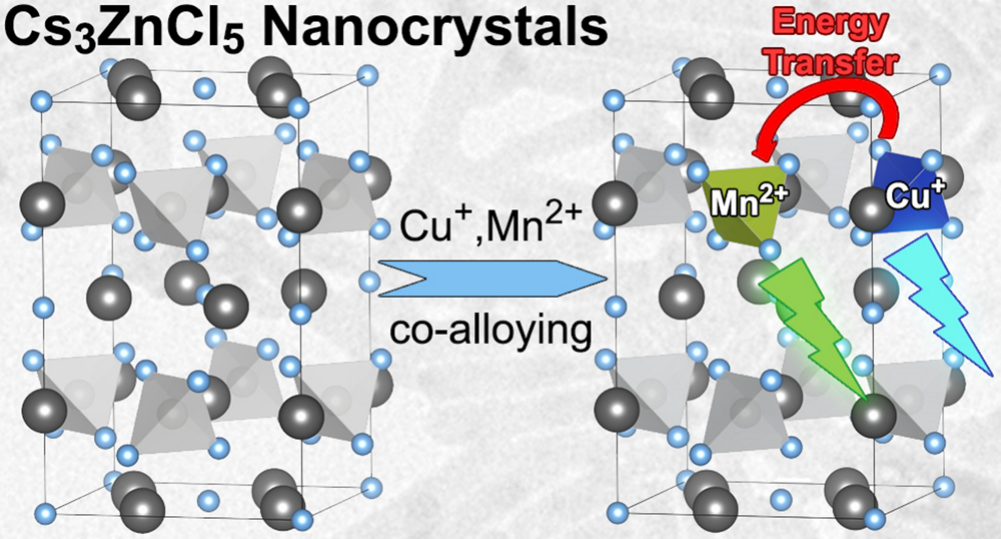

In this work, we report the hot-injection synthesis of Cs_3_ZnCl_5_ colloidal nanocrystals (NCs) with tunable amounts of Cu^+^ and Mn^2+^ substituent cations. All the samples had a rodlike morphology, with a diameter of ∼14 nm and a length of ∼30–100 nm. Alloying did not alter the crystal structure of the host Cs_3_ZnCl_5_ NCs, and Cu ions were mainly introduced in the oxidation state +1 according to X-ray photoelectron and electron paramagnetic resonance spectroscopies. The spectroscopic analysis of unalloyed, Cu-alloyed, Mn-alloyed, and Cu, Mn coalloyed NCs indicated that (i) the Cs_3_ZnCl_5_ NCs have a large band gap of ∼5.35 eV; (ii) Cu(I) aliovalent alloying leads to an absorption shoulder/peak at ∼4.8 eV and cyan photoluminescence (PL) peaked at 2.50 eV; (iii) Mn(II) isovalent alloying leads to weak Mn PL, which intensifies remarkably in the coalloyed samples, prompted by an energy transfer (ET) process between the Cu and Mn centers, favored by the overlap between the lowest (^6^A_1_ → ^4^T_1_) transition for tetrahedrally coordinated Mn^2+^ and the PL profile from Cu(I) species in the Cs_3_ZnCl_5_ NCs. The efficiency of this ET process reaches a value of 61% for the sample with the highest extent of Mn alloying. The PL quantum yield (QY) values in these Cu, Mn coalloyed NCs are lower at higher Mn contents. The analysis of the Mn PL dynamics in these samples indicates that this PL drop stems from inter-Mn exciton migration, which increases the likelihood of trapping in defect sites, in agreement with previous studies.

## Introduction

Lead halide perovskite nanocrystals (NCs) are characterized by narrow and tunable photoluminescence (PL) with a high quantum yield (QY). As such, they are being considered as potential candidates for various applications.^1−3^ However, despite their outstanding performance, perovskite NCs are poorly stable under air and are highly toxic because of the presence of lead and this severely limits their use in consumer products.^4,5^ Therefore, the development of lead-free halide perovskites or, more generally, alternative metal halide NCs with valuable optoelectronic properties is of great significance.^6−9^ Apart from a few exceptions, for example, Cu- and Sb-based metal halide NCs (e.g., Cs_3_Cu_2_I_5_ and Cs_3_Sb_2_Br_9_) which are characterized by a bright PL,^10,11^ most of the Pb-free NCs compounds reported so far per se have a relatively low PLQY or even no emission at all. Examples are Cs_2_SnI_6_,^12^ Cs_3_Bi_2_Br_9_,^13^ and double perovskite NCs (e.g., Cs_2_AgInCl_6_, Cs_2_AgBiBr_6_, and Cs_2_NaBiCl_6_)^14−16^ which feature PLQY values of the order of a few percentage points or Cs_2_ZnCl_4_,^17^ Cs_3_InCl_6_,^18^ Cs_2_TiBr_6_,^19^ and Cs_2_AgBiCl_6_^14^ NCs that basically have no PL. Hence, great efforts have been devoted to improve their optoelectronic properties using different means.^20−22^

If one considers the various strategies employed so far, doping or alloying, that is, the introduction of a target element ion into a host lattice, has led to the most promising results.^23−27^ Among the different substituents tested, Mn^2+^ and Cu^+^ cations were found to confer a blue, green, or orange emission to otherwise nonemissive or poorly emissive metal halide NCs.^17,28,29^ For example, Cu^+^ doping of nonluminescent Cs_2_ZnCl_4_ NCs and Cs_2_ZnBr_4_ powders yielded a bright blue PL with QY values as high as 50%,^17,30^ while Mn^2+^ doping in weakly luminescent Cs_2_AgInCl_6_ NCs and Cs_2_NaIn_0.75_Bi_0.25_Cl_6_ NCs led to orange emissions with ∼16% and 44.6% PLQY, respectively.^29,31^ In such systems, the Cu^+^ emission is caused by a transition from an excited 3d^9^4s^1^ state to a ground 3d^10^ state.^32,33^ Because of the energy-level splitting of the excited state, Cu^+^ ions are reported to exhibit a wide excitation and a broad emission range.^34^ For example, Cu^+^ ions in a CaS host feature a violet emission at 413 nm, while in Cs_2_ZnCl_4_ NCs, a blue emission at 486 nm, and in the BaS matrix, an orange emission at 585 nm.^33^ In those different host crystals, the excitation bands of Cu^+^ ions span the 260–400 nm range.^33^ On the other hand, the Mn^2+^ emission originates from atomic d-d electronic transitions and is characterized by a broad PL emission whose spectral position depends on the coordination geometry of the Mn^2+^ ions, on the strength of the crystal field and on the crystal field distortion:^35−40^ for Mn(II) ions coordinated by halide ions, a tetrahedral coordination yields a green emission,^35^ while an octahedral one leads to an orange–red or near-infrared emission.^38^

Because the Mn^2+^*d–d* transitions are spin- and parity-forbidden,^35^ sensitizers are often employed as codopants (examples are rare-earth ions, Bi^3+^, Sb^3+^, Cu^+^, etc.) to promote the Mn^2+^ dopants emission via an energy transfer (ET) process.^41−44^ ET has been proven to be an efficient way to boost light emission.^45−47^ Among the sensitizers, Cu^+^ ions are known to be good sensitizers in many luminescent materials:^48,49^ in aluminophosphate glasses, the incorporation of Cu^+^ was found to enhance the near-infrared emission of Nd^3+^;^50^ in borosilicate glasses, Cu^+^ doping was shown to cause a fivefold enhancement in the Mn^2+^ emission, and this was attributed to ET from Cu^+^ ions (absorbing in the UV region) to Mn^2+^;^51^ in Cu^+^/Eu^3+^ codoped borosilicate glasses, the emission intensity of Eu^3+^ increased by two times, and tunable emission was achieved due to ET from Cu^+^ to Eu^3+^ ions.^32^ The ET process from Cu^+^ to Mn^2+^ has been also reported in NaCl:Cu^+^,Mn^2+^ and CaS:Cu^+^,Mn^2+^ phosphors and in oxyfluoride glasses.^43,52,53^

**Scheme 1:**
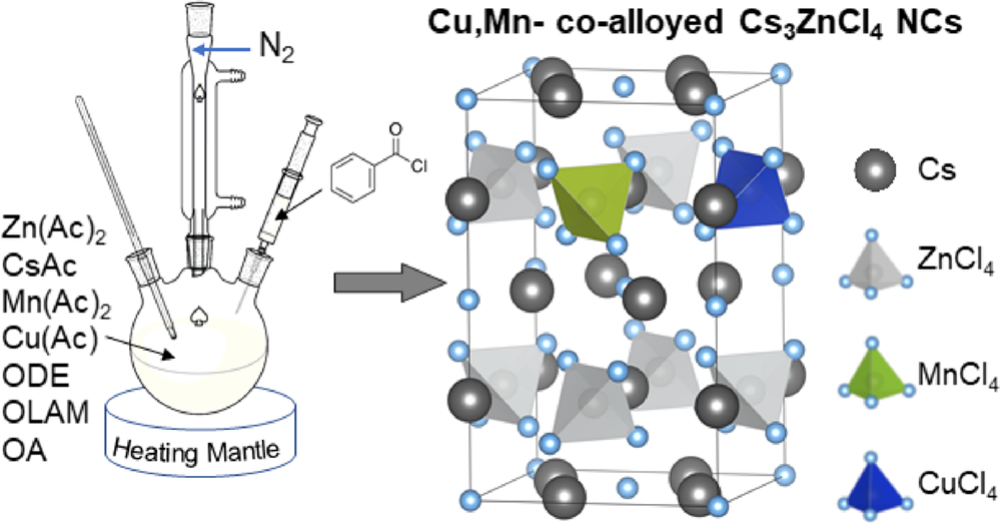
Synthesis and Structure of Cs_3_ZnCl_5_ Nanocrystals Alloying with Cu^+^ and/or Mn^2+^ Ions

Inspired by these studies, we investigated here if the emission from Mn^2+^ substituents can be sensitized by Cu^+^ cosubstituents in a wide band gap metal halide matrix. Because Cu^+^ ions can adopt only a tetrahedral coordination in metal halide structures (whereas Mn can have either a tetrahedral or an octahedral coordination),^17,54−56^ we selected Cs_3_ZnCl_5_ NCs as the host matrix, as the structure is composed of isolated ZnCl_4_ tetrahedra and thus being able to accommodate both Cu^+^ and Mn^2+^ substituents. In this work, we first developed a facile hot-injection method to synthesize Cs_3_ZnCl_5_ NCs with tunable amounts of Cu^+^ and Mn^2+^ substituents (Scheme 1). In the resulting NCs, which had a rodlike morphology, alloying did not alter the crystal structure, and Cu ions were mainly introduced in the oxidation state +1 according to X-ray photoelectron spectroscopy (XPS) and electron paramagnetic resonance (EPR) analyses. A systematic spectroscopic investigation of the NC samples revealed that the photophysical mechanism underlying the activation of the Mn luminescence is based on an ET scheme that connects Cu and Mn centers and that is prompted by the fair spectral overlap between the Cu PL and the lowest (^6^A_1_ → ^4^T_1_) Mn transition. The efficiency of this ET process, as extracted from time-resolved PL measurements, rapidly increases together with the Mn^2+^ content in the NCs, reaching the value of 61% for the highest Mn content. The PLQY values of these Cu, Mn coalloyed NCs was essentially constant (around 3%) apart for the sample featuring the highest Mn content (PLQY < 1%). The analysis of the Mn PL dynamics and the EPR spectra indicated that this QY drop stems from inter-Mn exciton migration, which increases the likelihood of trapping in defect sites, in agreement with previous studies on Mn-doped systems.^57^

## Experimental Section

### Chemicals

Zinc acetate (Zn(ac)_2_, 99.99%), cesium acetate (Cs(ac), 99.99%), copper(I) acetate (Cu(ac), 97%), manganese acetate (Mn(ac)_2_, 98%), 1-octadecene (ODE, 90%), oleylamine (OLAM, 98%), oleic acid (OA, 90%), hexane (anhydrous, 95%), ethyl acetate (99.9%), and benzoyl chloride (Bz-Cl, 98%) were purchased from Sigma-Aldrich. All chemicals were used without any further purification.

### Synthesis of Cs_3_ZnCl_5_ and Cu, Mn Coalloyed Cs_3_ZnCl_5_ NCs

Cs(ac) (0.2 mmol), a desired amount of Zn(ac)_2_, Mn(ac)_2_, and Cu(ac) (see Table 1), 2 mL of ODE, 1 mL of OA, and 2 mL of OLAM were loaded into a 50 mL three-necked flask and degassed under vacuum for 1 h at 130 °C. Then, the temperature was increased to 170 °C under N_2_, and 0.2 mL of Bz-Cl dispersed into 0.5 mL of degassed ODE was quickly injected. The system was quenched by an ice-water bath after 10 s. The crude solution was centrifuged at 4000 rpm for 10 min, and the precipitate was redispersed in 2 mL of hexane. The NCs were centrifuged again at 4000 rpm for 10 min, and the precipitate was dispersed in 1 mL of hexane. Eventually, 2 mL of ethyl acetate was added to the NCs-hexane dispersion and centrifugated at 6000 rpm for 5 min. The final precipitate was dispersed in hexane (1 mL) and stored in a N_2_-filled glovebox for further use. All the purification steps were performed in the glovebox.

**Table 1:** Molar Amounts of Zn, Mn, and Cu Precursors Employed in the Syntheses

sample	Zn(ac)_2_ mmol	Mn(ac)_2_ mmol	Cu(ac) mmol
9.8%Mn	0.168	0.043	0
12%Mn–1.7%Cu	0.168	0.043	0.02
9.6%Mn–2.2%Cu	0.168	0.043	0.048
9.8%Mn–6.4%Cu	0.168	0.043	0.096
3.4%Cu	0.24	0	0.048
4.5%Mn–4.6%Cu	0.204	0.036	0.048
30%Mn–7.9%Cu	0.096	0.087	0.048

### X-Ray Diffraction Characterization

X-ray diffraction (XRD) measurements were carried out on a PANanalytical Empyrean X-ray diffractometer equipped with a 1.8 kW Cu Kα ceramic X-ray tube and a PIXcel3D 2 × 2 area detector, operating at 45 kV and 40 mA. Specimens for XRD measurements were prepared by dropping concentrated NC solution onto a silicon zero-diffraction single crystal substrate. XRD patterns were collected under ambient conditions with a parallel beam geometry and symmetric reflection mode. Rietveld profile analysis was performed with the software package FullProf. The profile fit was performed by optimizing unit cell parameters, scale factor, background, anisotropic crystallite size (spherical harmonics model), and preferred orientation parameters. For refinement purposes, the composition of analyzed samples (undoped and 3.4% Cu^+^) was kept fixed to Cs_3_ZnCl_5_, as such dopant concentration is negligible for the technique.

### Elemental Analysis

Inductively coupled plasma optical emission spectroscopy (ICP-OES) was performed with an iCAP 6300 DUO ICP-OES spectrometer (ThermoScientific) to quantify the Cu/Zn and Mn/Zn ratios with a systematic error of about 5%. Samples for ICP-OES measurements were dissolved in 1 mL of aqua regia (HCl/HNO_3_ = 3/1(v/v)) overnight. Scanning electron microscopy (SEM) was performed on a HRSEM JEOL JSM-7500LA microscope with a cold field-emission gun (FEG), operating at 15 kV acceleration voltage. To evaluate the full elemental composition of these samples, energy-dispersive spectroscopy (EDX, Oxford instrument, X-Max, 80 mm^2^) was operated at 8 mm working distance, 15 kV acceleration voltage, and 15 sweep count.

### XPS Analysis

XPS analyses were carried out with a Kratos Axis Ultra^DLD^ spectrometer using a Mg Kα source, operated at 20 mA and 15 kV. The choice of using this X-ray source was to avoid the spectral overlapping of Cu 2p with the Cs MNN Auger lines we would have had if using instead the monochromatic Al Kα one. High-resolution analyses of Cu 2p peaks were carried out at a pass energy of 40 eV. The Kratos charge neutralizer system was used during data acquisition. Spectra have been charge-corrected to the main line of the C 1s spectrum (adventitious carbon) set to 284.8 eV. Spectra were analyzed using CasaXPS software (version 2.3.24).^58^

### Transmission Electron Microscopy Analysis

Transmission electron microscopy (TEM) analyses were carried out on a JEOL JEM-1400Plus microscope with a thermionic gun (LaB_6_ crystal) working at an acceleration voltage of 120 kV. The samples for TEM measurement were prepared by dropping dilute NC hexane solutions onto carbon film-coated 200 mesh copper grids.

### Optical Measurements

Absorption spectra were recorded using a Varian Cary 50 ultraviolet–visible absorption spectrophotometer. The steady-state PL and PL excitation (PLE) spectra were measured on a Varian Cary Eclipse spectrophotometer. Time-resolved PL experiments were conducted by exciting the samples with a frequency quadrupled Q-switched Nd:YAG laser at 4.66 eV collecting with a Hamamatsu R943-02 time-correlated single-photon counting unit coupled to an Oriel Instruments Cornerstone 260 monochromator. The PL efficiencies were measured by comparing the PL intensity of the NCs, and the emission from Quinine Sulfate dissolved in 0.5 M H_2_SO_4_ solution was used as the standard reference dye, following the method described in the work of Resch-Genger and co-workers.^59^ All the optical measurements were conducted at room temperature on hexane dispersions of NCs kept under protective atmosphere.

### Radio Luminescence

The NC samples were excited by X-ray irradiation through a beryllium window, using a Philips 2274 XRD tube (with a tungsten target) operated at 20 kV. At this operating voltage, X-rays are produced by the *Bremsstrahlung* mechanism because of the impact of electrons generated through a thermionic effect and accelerated onto the tungsten target, resulting in a continuous distribution of energies peaked around ∼7 keV. The spectra were collected at room temperature with a homemade apparatus featuring a liquid nitrogen-cooled, back-illuminated, and UV-enhanced, CCD detector (Jobin Yvon Symphony II) coupled to a monochromator (Jobin Yvon Triax 180) equipped with a 100 grooves/mm grating as the detection system. The spectra were corrected for the spectral response of the acquisition system. All the RL measures were performed on NCs casted on Al_2_O_3_-coated aluminum substrates.

### EPR Measurements

Solution **s**amples were prepared inside a glovebox and loaded into a suprasil EPR quartz tube sealed with a tip-off manifold to allow a transfer into the EPR cavity without exposure to air. CW-EPR spectra were recorded at room temperature on a Varian spectrometer coupled to a Bruker super-High Q cavity (ER 4122SHQE). Typical experimental parameters were modulation frequency 100 kHz, modulation amplitude: 5 Gauss, microwave power: 5 mW. Spectra were simulated with the Easyspin Toolbox.^60^

### Computational Methodology

We built our computational models by starting from a bulk Cs_3_ZnCl_5_ tetragonal cell, which was then doped (i) with one Mn(II) ion (replacing one Zn(II) ion); (ii) with one Cu(I) ion (replacing one Zn(II) ion and charge-balanced by removing a chloride ion, resulting in the CuCl_3_ unit); and (iii) with both a Mn(II) ion and a Cu(I) ion located either in neighboring or non-neighboring positions. We also decided to choose a large 2 × 2 × 2 supercell to obtain doping concentrations of about 3%, in line with the experiments. For all the abovementioned supercell structures, atomistic calculations were performed at the Γ point of the 2 × 2 × 2 supercell, considering that the disconnection between the tetrahedral units prevents the formation of dispersive band structures. Both atomic positions and cell parameters were relaxed at the density functional theory (DFT) level using the PBE exchange correlation functional^61^ and a double-ζ basis set plus polarization functions (DZVP) on all atoms,^62^ as implemented in the CP2K 6.1 package.^63^ Scalar relativistic effects were incorporated as effective core potentials.^64^

## Results and Discussion

**Figure 1:**
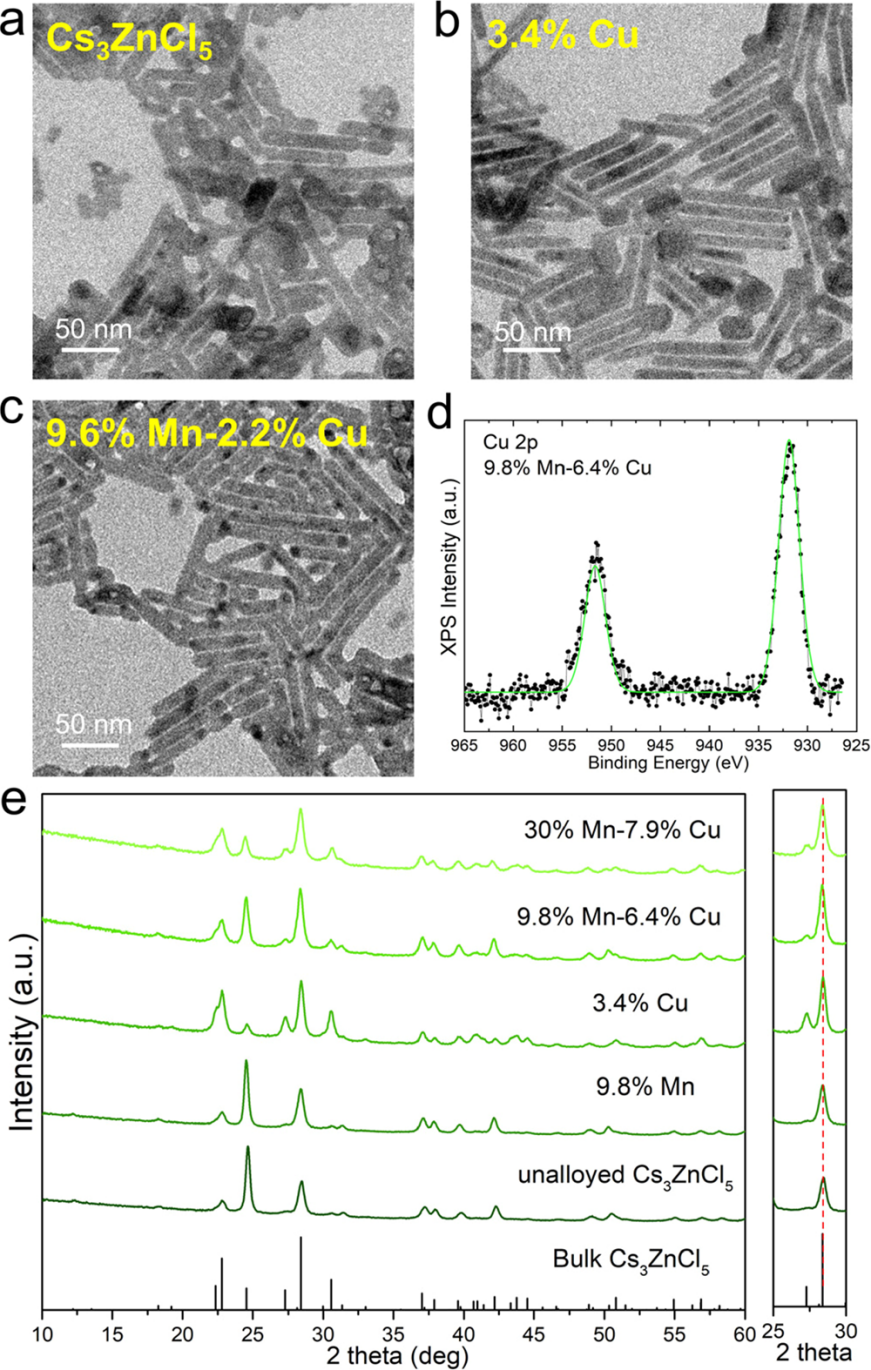
(a–c) TEM images of unalloyed, 3.4%Cu and 9.6%Mn–2.2%Cu NC samples. (d) A selected region of the XPS spectra of the 9.8%Mn–6.4%Cu sample. (e) XRD patterns of representative samples with corresponding reflections of bulk Cs_3_ZnCl_5_ (ICSD number 240876) with the magnification of the 25–30° 2 theta range.

The first step in this work was to develop a colloidal synthesis of Cs_3_ZnCl_5_ NCs. This was based on a hot-injection method in which metal carboxylate precursors, namely, Cs and Zn acetates, were mixed under a N_2_ atmosphere with degassed OA, oleylamine, and octadecene and heated up to 170 °C, after which benzoyl chloride was swiftly injected to trigger the NCs nucleation and growth (Scheme 1). The NCs feature a rodlike shape (Figure 1a) and the tetragonal Cs_3_ZnCl_5_ bulk structure (ICSD number 240876) (Figure 1e, bottom pattern). We then synthesized various samples of Cs_3_ZnCl_5_ NCs alloyed with Cu and Mn. This was done by exploiting the same reaction scheme for the unalloyed NCs, with the addition of Mn(II) and Cu(I) acetates as the substituent precursors (see the Experimental Section). The ratios of Mn/Zn and Cu/Zn were varied in a way to explore a wide range of alloying levels for Mn^2+^ (up to 30%) because isovalent alloying with Mn^2+^ is not expected to modify the crystal structure of Cs_3_ZnCl_5_, even at a high Mn content.^65^ On the other hand, we kept low levels of alloying with Cu^+^ (up to ∼8%) because this type of aliovalent replacement might induce a phase transition in the Cs_3_ZnCl_5_ host matrix past a certain threshold. The composition of the NC samples and alloying levels, expressed as Mn/Zn and Cu/Zn percentages, were measured via SEM–EDX and ICP-OES analyses (Table 2).

**Table 2:** Elemental Analyses Performed via SEM–EDS and ICP-OES^a^

sample name	SEM–EDS analysis		ICP-OES analysis	
	composition	Mn (%)	Mn (%)	Cu (%)
9.8%Mn	Cs_3.24_Zn_0.90_Mn_0.10_Cl_5.62_	10.8	9.8	0.0
12%Mn–1.7%Cu	Cs_3.10_Zn_0.89_Mn_0.11_Cl_5.79_	12.3	12.0	1.7
9.6%Mn–2.2%Cu	Cs_3.13_Zn_0.91_Mn_0.09_Cl_6.01_	9.9	9.6	2.2
9.8%Mn–6.4%Cu	Cs_3.11_Zn_0.92_Mn_0.08_Cl_5.86_	9.1	9.8	6.4
3.4%Cu	Cs_3.10_ZnCl_5.64_	0.0	0.0	3.4
4.5%Mn–4.6%Cu	Cs_3.05_Zn_0.96_Mn_0.04_Cl_5.52_	4.0	4.5	4.6
30%Mn–7.9%Cu	Cs_3.15_Zn_0.77_Mn_0.23_Cl_5.85_	30.2	30.0	7.9

a The Mn and Cu percentages are expressed as Mn/Zn and Cu/Zn, respectively.

To investigate the oxidation state of Cu ions in our samples, we carried out XPS analysis on the 9.8%Mn–6.4%Cu sample, which bears one of the highest Cu contents of the series. This choice of the sample was motivated by the fact that as emerged from our previous work on Cu-alloyed Cs_2_ZnCl_4_ NCs,^17^ Cu^2+^ species might be found in samples with high Cu-alloying levels. We examined in detail the Cu 2p region: the position of the Cu 2p peak (931.9 ± 0.2 eV) in Figure 1d, close to the reported peak for Cu 2p_3/2_ at 932 eV binding energy for Cu(Ι) compounds,^66,67^ pointed to Cu cations being only in the +1 oxidation state. Moreover, the collected XPS data did not show the typical satellites reported for Cu(II) compounds, usually seen in the 938–946 eV and 962–964 eV ranges.^67^ Hence, we conclude that Cu is present in the NCs only with oxidation state +1, and this is also confirmed by EPR, as discussed later in detail. As a note, the presence of Cu^+^ ions as alivoalent substituents raises the issue of how charge is compensated. This is most likely achieved by the presence of anion/cation vacancies, by charged surface ligands, or both.

TEM analysis revealed that all the samples (including the unalloyed Cs_3_ZnCl_5_ NCs and the various alloyed samples) consist of NCs having a rodlike shape with a diameter of ∼14 nm and a length of 30∼100 nm (see Figure 1a–c and S2a). XRD patterns of all the NC samples matched well with the tetragonal Cs_3_ZnCl_5_ bulk structure (ICSD number 240876), with no presence of impurities (Figures 1e, S1, and S2b–d). Upon careful inspection, no XRD peaks shifts were observed after the incorporation of the Cu^+^ and/or Mn^2+^ substituents (see the right panel of Figure 1c). This was attributed to the similarity in ionic radii among tetrahedrally coordinated Cu^+^, Mn^2+^, and Zn^2+^ ions (0.6, 0.66, and 0.6 Å, respectively).^68^

The intensity mismatch observed in the diffraction peaks of Figures 1e and S1 can be entirely attributed to different degrees of preferred orientation for the different samples. To demonstrate this, we performed a Rietveld refinement of two patterns including preferred orientation and anisotropic shape effects but keeping the composition and structure of the material fixed (Figure S3). The analysis confirmed that a satisfactory convergence with the experimental data can be reached without considering any stoichiometry-related effects. Indeed, doping on the order of 4.3%, as reported in the second pattern of Figure S3, is too small to cause important effects on the intensity of the diffraction peaks and is probably below the detection limit for nanocrystalline samples, for which morphology and orientation effects are more impactful.

**Figure 2:**
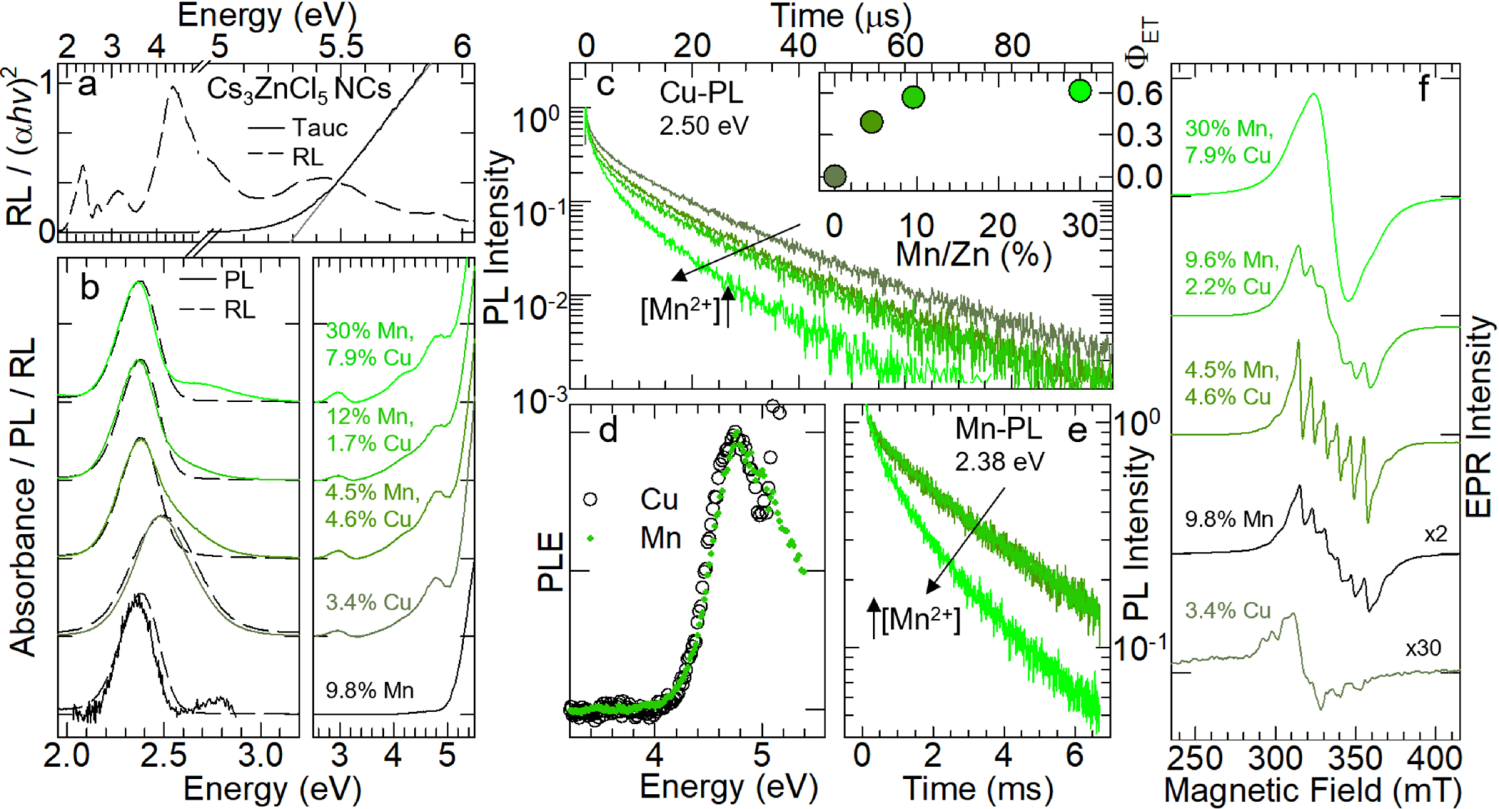
(a) Tauc plot (solid line) and RL spectrum (dashed line) of the unalloyed Cs_3_ZnCl_5_ NCs. The gray solid line represents the fit with the theoretical absorption profile of a semiconductor with a direct permitted energy gap; the intercept with the abscissa axis corresponds to the semiconductor’s forbidden gap energy. (b) Absorption (right panel), PL, and RL (left panel) spectra for Cu, Mn (co)-alloyed Cs_3_ZnCl_5_ NCs at different substituent concentrations, as indicated in the figure. All the spectra were normalized and vertically shifted for clarity. (c) Normalized Cu PL decay of Cu, Mn coalloyed NC samples with increasing Mn^2+^ content. Inset: the corresponding energy transfer quantum yields as defined in the main text. The Mn concentrations are reported as Mn/Zn (%) molar ratios according to Table 2. (d) Time-gated normalized PLE spectra for 4.5%Mn, 4.6%Cu coalloyed Cs_3_ZnCl_5_ NCs collected at the Cu (empty circles) and Mn PL (filled circles) maxima. (e) Normalized Mn PL decays of Cu, Mn coalloyed NC samples with increasing Mn^2+^ content. All the PL and PL decays were collected using 4.66 eV (266 nm) pulsed excitation modulated at 15 Hz. The PL emission of the 9.8%Mn sample was measured by employing at 5.4 eV source. (f) Room temperature EPR spectra of 3.4%Cu alloyed, 9.8%Mn alloyed, and Cu, Mn coalloyed NC samples. The spectra are offset for clarity.

We next investigated the optical properties of all the NC samples by a combination of optical spectroscopy and radioluminescence (RL), the latter enabling us to excite the Cs_3_ZnCl_5_ NC matrix directly. The unalloyed Cs_3_ZnCl_5_ NCs have a wide gap of ∼5.35 eV as extracted from the respective Tauc plot shown in Figure 2a. Excitation of such a large band gap system above 5.4 eV did not lead to any appreciable PL. Instead, the excitation with soft X-rays produced a structured RL spectrum with a broad peak close to the band-edge energy and lower energy contributions, possibly originating from emissive defect states. The effect of alloying of Cs_3_ZnCl_5_ NCs with either Cu, Mn, or both on their optical properties is seen in Figure 2b, which reports the optical absorption, PL, and RL spectra of the corresponding samples. The NCs alloyed with only Mn ions (9.8%Mn sample in Figure 2b) retained the absorption profile of the unalloyed Cs_3_ZnCl_5_ NCs, in agreement with the forbidden nature of the Mn *d–d* transitions. When optically excited at 5.4 eV, these samples exhibited a weak Mn PL peak at 2.37 eV originating from the spin forbidden ^4^T_1_-^6^A_1_ transition of excited Mn^2+^ in tetrahedral coordination, as documented in previous reports on the luminescence of Cs_3_MnBr_5_ powders^69^ and Mn-doped Cs_3_Zn(Cl/Br)_5_ single crystals,^70^ indicating that the Mn emission can be activated via excitation of the host matrix. The NCs alloyed with Cu^+^ ions only (3.4%Cu sample in Figure 2b) were characterized by an absorption feature at ∼4.8 eV with a minor contribution at ∼2.9 eV, not present in the host NCs and which were previously ascribed to localized Cu^+^ states.^17^ Optical excitation of this sample at 4.66 eV, that is, within the Cu-related absorption peak, resulted in a Stokes-shifted Cu PL band at 2.50 eV, consistent with the recombination of excitons that are self-trapped in tetrahedral Cu^+^ centers.^17^ Crucially, in Cu, Mn coalloyed NCs, the direct excitation of the Cu^+^ states activated the Mn PL at 2.37 eV, which became dominant even at the lowest level of Mn^2+^ alloying (4.5%Mn–4.6%Cu sample). The Cu PL progressively diminished when further increasing the Mn^2+^ content inside the NCs (Figure 2b, samples 12%Mn–1.7%Cu and 30%Mn–7.9%Cu). This suggests that the two centers (Cu and Mn) are connected by an ET process prompted by the fair overlap between the lowest (^6^A_1_ → ^4^T_1_) excitation peak for tetrahedral coordinated Mn^2+^ and the Cu PL profile.^69,71^ The RL of all NCs (Figure 2b) always matched with the corresponding PL, meaning that the Cu^+^ and Mn^2+^ states act as recombination centers in both scintillation and photoluminescence processes. Interestingly, we notice that in Mn, Cu coalloyed NCs, the Cu-related emission appears only in the PL measurements, whereas it is absent in the corresponding RL spectra. This behavior is probably due to the different photophysical mechanisms that underlie the PL and RL processes. Specifically, under optical excitation, the activation of the Mn^2+^ centers likely occurs upon an ET mechanism from Cu^+^ excited states, thus leading to a residual Cu PL contribution that is inversely related to the efficiency of the ET process. On the other hand, the RL involves a more complex chain of events (e.g., primary interactions with the ionizing radiation, secondary events like further ionization and excitation of the whole medium, trapping and detrapping processes during the carrier migration to recombination sites) with no obvious carrier relaxation pathways. Within this framework, the absence of Cu RL in Cu, Mn coalloyed NCs might be due to the existence of a more efficient excitation channel for Mn^2+^ centers, involving high-energy states and completely decoupled from Cu. Our observations on Mn-only alloyed NCs corroborates this picture: indeed, although their PL is barely detectable (see also the signal to noise ratio of the PL spectrum in Figure 2b), the corresponding RL intensity is comparable to that of the other NCs, thus suggesting that the Mn radioluminescence can be directly excited upon X-ray excitation.

Further indications about the ET mechanism came from time-resolved PL measurements in Figure 2c showing the acceleration of the Cu PL dynamics, which evolves from ∼3.5 μs in the 3.4%Cu sample to ∼1.3 μs in the 30%Mn–7.9%Cu sample (that is, the sample featuring the highest Mn content). For this series of samples, the Cu PL dynamics were well reproduced by a stretched exponential function:
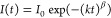
where *I*_0_ is the zero-delay PL intensity, *k* is the decay rate, and β (∼0.47) is the stretching factor, which is found to be weakly influenced by the increase of Mn^2+^ alloying. From the evolution of the Cu PL decay kinetics, we quantified the ET efficiency through the following expression:
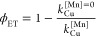
where *k*_Cu_^[Mn]^ is the decay rate of the Cu PL at different levels of Mn alloying (indicated as [Mn]). As shown in the inset of Figure 2c, ϕ_ET_ reaches over 61% for the largest Mn content. Further confirmation of the key role of Cu in the activation of the Mn PL came from the PLE spectrum (Figure 2d) of a NC sample exhibiting both Mn- and Cu-related emissions (4.5%Mn–4.6%Cu sample). By operating in time-gated mode, it was possible to selectively monitor either the faster μs-long Cu PL or the slower ms-lived Mn PL. Both acquisition modes returned the same excitation peak at 4.75 eV in close match with the Cu absorption profile, further confirming that in our NCs, the Mn PL is activated upon excitation of the Cu centers and not via direct excitation of Mn^2+^ transitions.

The measured PLQY was essentially constant at ∼3% across the whole set of alloyed NC, except for the heaviest Mn-alloyed sample (30%Mn–7.9%Cu) that had a PLQY < 1%, and the Mn-only alloyed (9.8%Mn sample), which featured a barely detectable PL. Because the emission from all the Cu, Mn coalloyed Cs_3_ZnCl_5_ NC samples was dominated by the Mn PL, we extended our spectroscopic investigation to the Mn PL dynamics to identify the cause of such a lower PLQY value at the highest Mn alloying levels. As shown in Figure 2e, the lowest Mn-alloyed NCs (4.5%Mn–4.6%Cu sample) exhibited the characteristic single-exponential decay of nearly isolated Mn^2+^-centers with rate *k*^Mn^∼0.3 ms^–1^. At higher Mn contents, the Mn PL dynamics markedly accelerated and became multiexponential (with stretching factor β ∼ 0.58), accompanied by a drop of the respective PL efficiency. These are typical spectroscopic signatures of the activation of inter-Mn exciton migration promoting PL quenching in defect sites, in agreement with previous reports.^57,71,72^ EPR spectra of Cu-alloyed Cs_3_ZnCl_5_ NCs with increasing Mn content corroborate this picture, showing the transition from the typical signature of nearly isolated Mn^2+^ ions (i.e., the six evenly spaced resonances because of the ^55^Mn hyperfine transitions) in lightly alloyed particles (4.5%Mn–4.6%Cu sample) into coupled Mn^2+^ centers with increasing alloying level resulting from the magnetic dipolar broadening between Mn spin centers (Figure 2f). The relatively large isotropic hyperfine splitting *A* ∼230 MHz obtained from the EPR spectrum for the sample 4.5%Mn–4.6%Cu sample suggests that paramagnetic Mn ions are preferentially located at the surface of the NCs.^73^ The intensity and the position of the EPR spectrum of the Cu-only alloyed NCs (3.4%Cu sample) indicate that Cu is mostly introduced as Cu^+^, in agreement with XPS analysis.^17^ Therefore, it is not possible to assess the location of the majority of Cu dopants inside the NCs. On the other hand, the very weak EPR signal observed in the Cu-only sample is due to a tiny fraction of Cu^2+^ ions (see also Figure S4) which are therefore expected to play a negligible role, if any, to the ET process. The EPR spectrum recorded for the Mn-alloyed sample did not show significant changes neither in the hyperfine splitting nor in the linewidth if compared with the Cu, Mn coalloyed NCs with similar Mn content (i.e., 9.6%Mn–2.2%Cu sample), thus corroborating our XPS findings that Cu ions are mainly introduced in the oxidation state +1.

**Figure 3:**
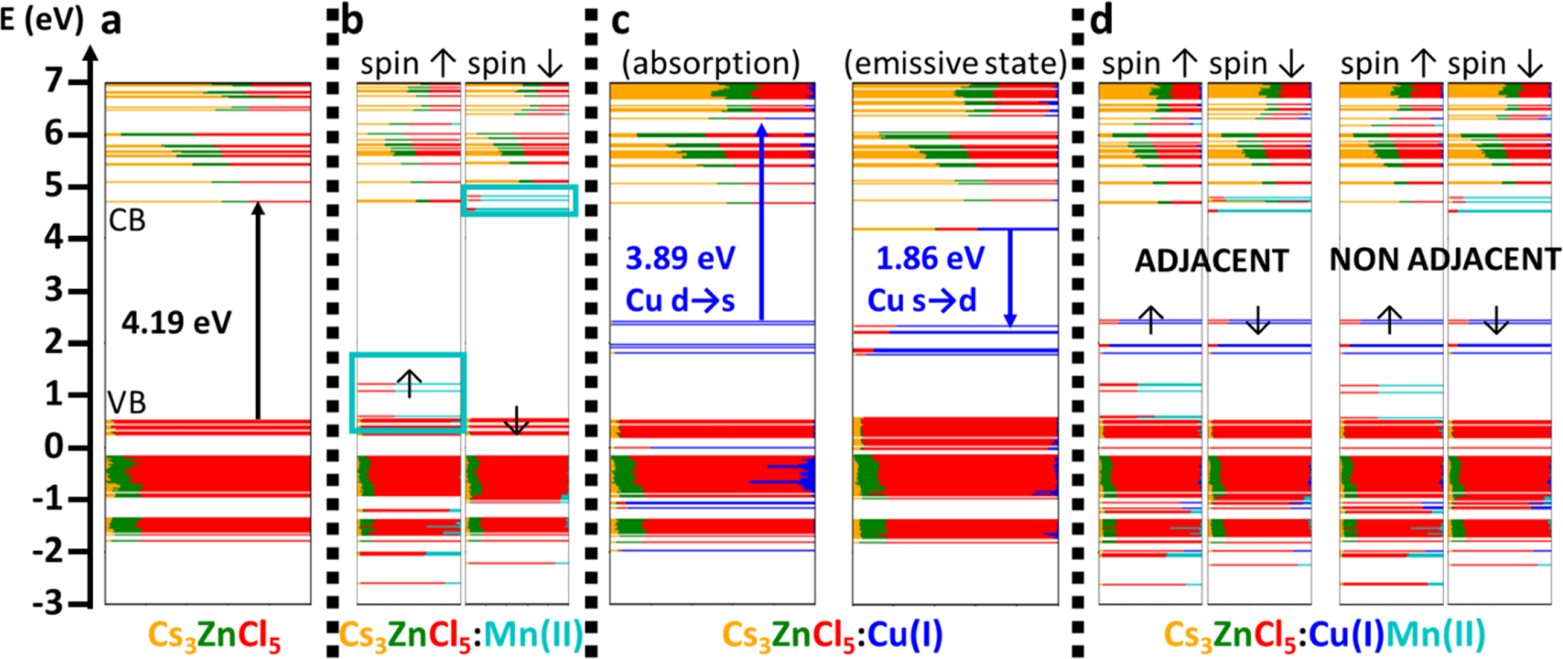
Electronic structure computed at the DFT/PBE level of theory at the Γ point of a 2 × 2 × 2 supercell for (a) Cs_3_ZnCl_5_, (b) Cs_3_ZnCl_5_:Mn(II), (c) Cs_3_ZnCl_5_:Cu(I), and (d) Cs_3_ZnCl_5_:Cu(I)Mn(II). In the latter case, substituent cations are located either in adjacent or nonadjacent positions. Cell parameters and ionic positions of all systems were relaxed in the ground state, a closed-shell singlet for (a, c) and a sextuplet for (b, d). The sextuplet spin magnetization of Mn(II) in the ground state results in the broken spin up and spin down configurations of panels (b) and (d). Panel (c) also contains the electronic structure of the Cu(I) emissive state, simulated by relaxing cell parameters and ionic positions in the lowest triplet (excited) state followed by a single point calculation in the singlet (ground) state at this new geometry.

To unravel the origin of the ET mechanism, we studied the role of Cu-alloying, Mn alloying, and Cu, Mn coalloying at the DFT/PBE level of theory. We started our investigation by looking at the electronic structure of the undoped Cs_3_ZnCl_5_ system at the Γ point, as illustrated in Figure 3a, and found a band gap of ∼4.2 eV. Unsurprisingly, this value is lower than the one observed experimentally as PBE functionals tend to underestimate the band gap. This correction is ∼0.5–1 eV for 0D systems,^17^ a value that we took as reference in the following discussion. We then replaced one Zn(II) ion by one Mn(II) ion into the Cs_3_ZnCl_5_ supercell, thus obtaining a Mn alloying concentration of about 3%. Owing to the *d*^5^ configuration of Mn(II) in the local MnCl_4_ unit, the electronic structure of this system presents a sextuplet spin magnetization, as depicted in Figure 3b, corresponding to the ^6^A_1_ ground state with both occupied and unoccupied Mn 3*d* orbitals lying in the band gap above the valence band (VB) and below the conduction band (CB) of the Cs_3_ZnCl_5_ matrix, respectively. We also attempted to compute the ^4^T_1_ state to study the ^4^T_1_ → ^6^A_1_ emission mechanism, but it turned out to be challenging due to the presence of a manifold of nearly degenerate quartet states that prevent convergence to the correct one. We thus computed the most stable quartet state to set it as a lower bound to the emission energy and a reference for the discussion below. After the structural relaxation, the computed emission from this state was at 1.70 eV, in qualitative agreement with the typically measured Mn(II) emission at 2.35 eV, also considering the DFT band gap underestimation.

We then proceeded by simulating the Cu(I)-alloying Cs_3_ZnCl_5_ system, obtained by substituting one Zn(II) ion with one Cu(I) ion and removing a chloride ion attached to it, thus forming a CuCl_3_ unit, to preserve the charge neutrality. As shown in Figure 3c, after structural relaxation, there are two main changes in the electronic structure: (i) the occupied 3d orbitals of Cu(I) emerge as five orbitals localized above the VB; (ii) the empty 4s orbital of Cu(I) appears deep inside the CB, in a heavily mixed configuration with both the 6s orbitals of Cs and 4s orbitals of Zn from the surrounding matrix. The calculated energy gap between these states is around 3.9 eV, that is, ∼0.3 eV below that of the matrix, in line with the absorption shoulder observed at ∼4.8 eV in the experiments on the Cu-doped system. To account for the emission mechanism, we computed the lowest triplet state, a common strategy to mimic the behavior of the lowest (singlet) excited state.^17,74^ Upon structural relaxation, the CuCl_3_ unit undergoes an important transformation from a trigonal planar configuration (in the ground state) to a pyramidal one (in the triplet state), as already reported for Cu(I)-doped Cs_2_ZnCl_4_ systems.^17^ In this configuration, the Cu(I) 4s-based molecular orbital drops inside the band gap of Cs_3_ZnCl_5_, thus generating a localized self-trapped exciton with an energy of ∼1.86 eV (Figure 3c, right panel). Assuming the typical DFT underestimation, this is in qualitative agreement with the broad emission at ∼2.5 eV observed in the experiments.

Finally, we investigated the effect of coalloying the Cs_3_ZnCl_5_ system by replacing two Zn(II) ions with one Cu(I) and one Mn(II) ions to form both CuCl_3_ and MnCl_4_ units in the supercell, either at adjacent or at distant positions (Figure 3d). Our DFT calculations revealed a negligible difference in energy between the two probed configurations (difference of ∼0.13 kcal/mol) which also present very similar electronic features. The only difference here lies on a slight larger mixing of the quasi-degenerate Mn 4s and Cu 4s-based states in the CB, occurring only in case of neighboring CuCl_3_ and MnCl_4_ units (Figure 3d). However, this negligible overlap, which only occurs in the CB, suggests that the ET is likely not mediated by phonons.

## Conclusions

In conclusion, we have developed a simple hot-injection method for the colloidal synthesis of Cs_3_ZnCl_5_ NCs featuring a favorable crystalline and wide gap energy structure to accommodate optically active Cu^+^ and Mn^2+^ substituents in tetrahedral lattice sites at concentrations as high as 7.9 and 30%, respectively. The introduction of Cu^+^ substituents activated a cyan emission at 2.5 eV that was largely Stokes-shifted from its main absorption feature (by 2.3 eV), while the Mn, Cu coalloying resulted in an effective (efficiency up to 61%) ET scheme, which prompted a green Mn PL in NCs that otherwise would be almost nonluminescent. The PLQY was found to be constant (∼3%) across different substituent molar ratios except for the most Mn-alloyed sample where the efficiency decreased below 1%. The origin of such efficiency drop was investigated with time-resolved PL and EPR measurements and was ascribed to excitation migration toward defect sites promoted by inter-Mn coupling occurring at increasing Mn^2+^ contents. Our DFT calculations indicated that the ET occurs from the Cu(I) emissive donor state (computed at 1.86 eV) and the Mn acceptor state (computed at 1.70 eV) and is not mediated by phonons. This work expands our knowledge on alloying in metal halide nanocrystals and on the ET process involving substituent species and suggests that similar ET schemes can be adopted in many other metal halide nanocrystal hosts.

## Supporting Information Available

XRD data, TEM images, and simulated EPR spectra (PDF)
