# Supplementary material for: Cu+ → Mn2+ Energy Transfer in Cu, Mn Coalloyed Cs3ZnCl5 Colloidal Nanocrystals
Source: Chem Mater. 2022 Sep 20;34(19):8603–12. doi: 10.1021/acs.chemmater.2c01578 (PMC9558458; doi:10.1021/acs.chemmater.2c01578)
Supplement: Supplementary file 1 — cm2c01578_si_001.pdf [file cm2c01578_si_001.pdf]

Supporting Information for:

$Cu^{+} \rightarrow Mn^{2+}$ Energy Transfer in Cu, Mn- co-alloyed Cs_3ZnCl_5 Colloidal Nanocrystals

Ying Liu[1,2‡], Matteo L. Zaffalon[3‡], Juliette Zito[2,4], Francesca Cova[3], Fabrizio Moro[3], Marco Fanciulli[3], Dongxu Zhu[2], Stefano Toso[2,5], Zhiguo Xia[6], Ivan Infante[2], Luca De Trizio[2*], Sergio Brovelli[3*], Liberato Manna[2*]

[1]Key Laboratory of Materials Physics of Ministry of Education, School of Physics and Microelectronics, Zhengzhou University, Daxue Road 75, Zhengzhou 450052, China

[2]Istituto Italiano di Tecnologia, via Morego 30, Genova IT-16163, Italy

[3]Dipartimento di Scienza dei Materiali, Università degli Studi Milano-Bicocca, via R. Cozzi 55, Milano IT-20125, Italy

[4]Dipartimento di Chimica e Chimica Industriale, Università degli Studi di Genova, Via Dodecaneso 31, 16146 Genova, Italy

[5]International Doctoral Program in Science, Università Cattolica del Sacro Cuore, 25121 Brescia, Italy

[6]The State Key Laboratory of Luminescent Materials and Devices, Guangdong Provincial Key Laboratory of Fiber Laser Materials and Applied Techniques, School of Physics and Optoelectronics, South China University of Technology, Guangzhou, 510641, P. R. China

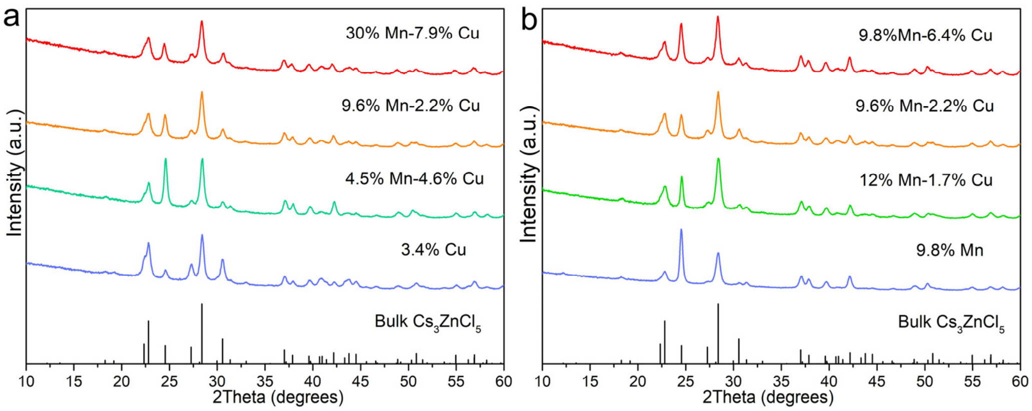

Figure S1. (a, b) XRD patterns of unalloyed and Cu, Mn (co)-alloyed Cs_3ZnCl_5 NC samples with corresponding reflections of bulk Cs_3ZnCl_5 (ICSD number 240876).

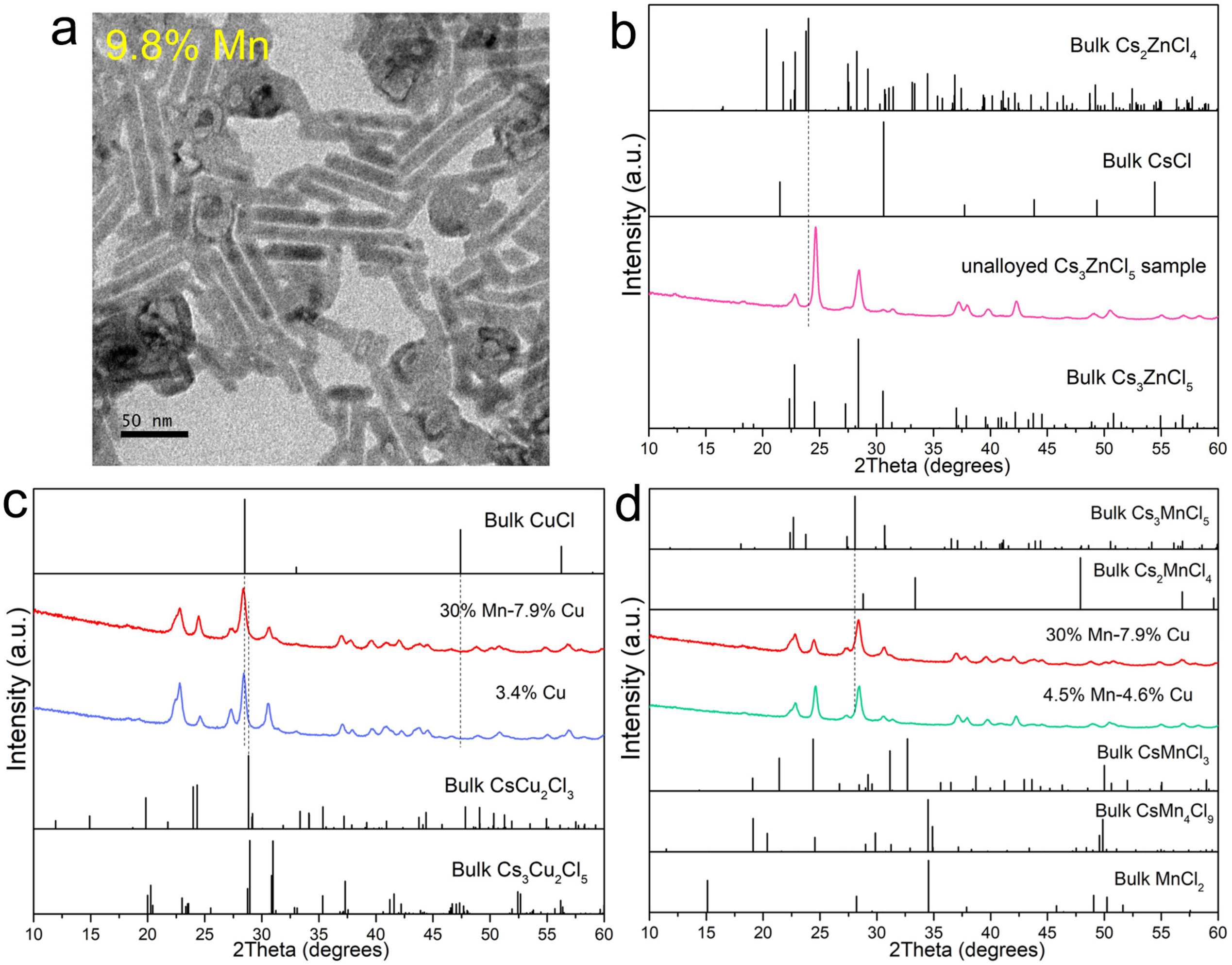

Figure S2. (a) TEM images of 9.8% Mn alloyed Cs_3ZnCl_5 NCs. (b-d) Comparison of XRD patterns between obtained samples and possible phases containing Cu^+ and Mn^{2+} ions. (b) XRD patterns of unalloyed Cs_3ZnCl_5 NC samples with corresponding reflections of bulk Cs_3ZnCl_5 (ICSD number 240876) and possible phases CsCl (ICSD number 622367) and Cs_2ZnCl_4 (ICSD number 6062). (c) XRD patterns of 3.4% Cu alloyed and 7.9% Cu, 30% Mn alloyed Cs_3ZnCl_5 NC samples with possible phases $CsCu_2Cl_3$ (ICSD number 14201), $Cs_3Cu_2Cl_5$ (ICSD number 22951), CuCl (ICSD number 73255). (d) XRD patterns of 4.6% Cu, 4.5% Mn alloyed and 7.9% Cu, 30% Mn alloyed Cs_3ZnCl_5 NC samples compared with possible phases $MnCl_2$ (ICSD number 33752), $CsMn_4Cl_9$ (ICSD number 34750), $CsMnCl_3$ (ICSD number 2525), Cs_2MnCl_4 (ICSD number 73253) and Cs_3MnCl_5 (ICSD number 24).

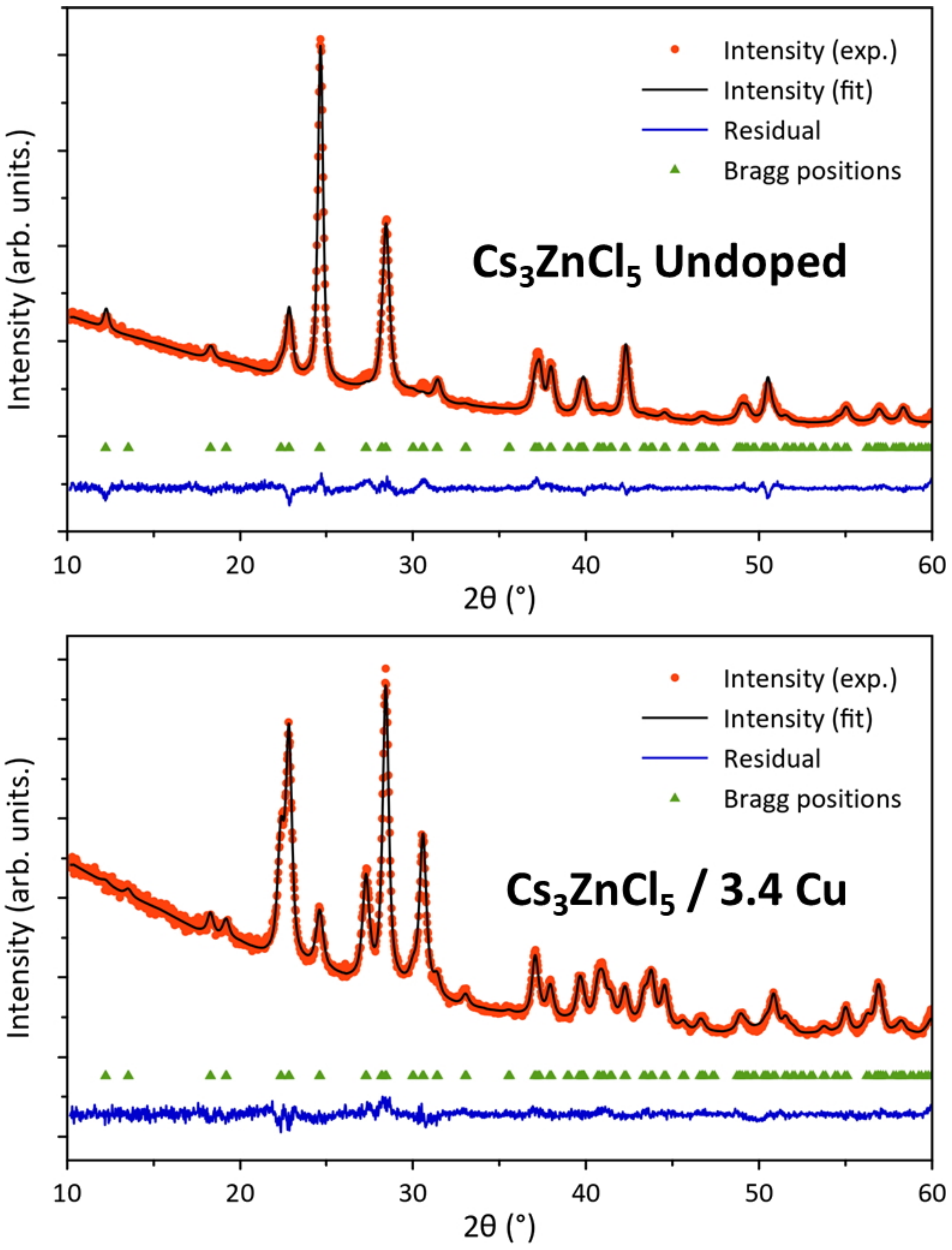

Figure S3. Rietveld refinement of XRD patterns for unalloyed Cs_3ZnCl_5 and 3.4% Cu alloyed Cs_3ZnCl_5 samples.

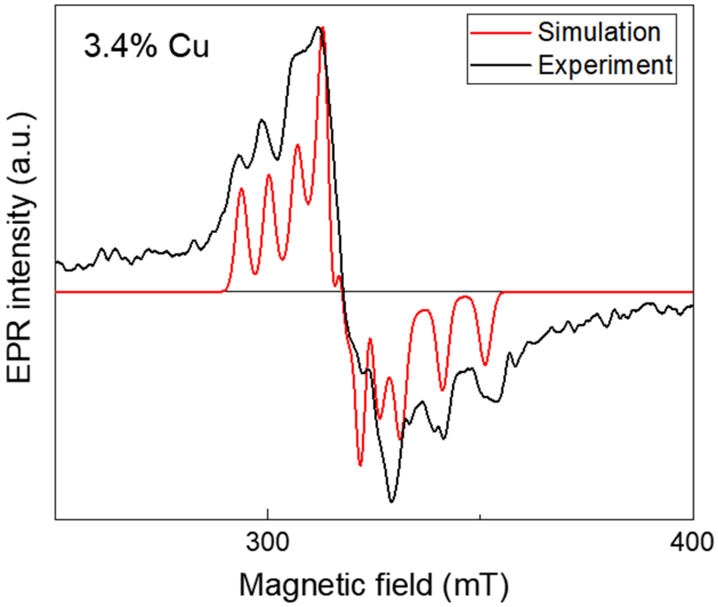

Figure S4. The EPR spectrum of Cu-alloyed NCs (3.4% Cu sample) and the simulated EPR spectrum - as indicated in figure - for Cu^{2+} centers using the EasySpin Toolbox (see the Methods section) and the following fitting parameters: $^{63}Cu^{2+}$ and $^{65}Cu^{2+}$ with natural abundances, g-values [g_{xx}, g_{yy}, g_{zz}] = [1.996, 2.1, 2.21], and hyperfine interactions [A_{xx}, A_{yy}, A_{zz}] = [270, 100, 200] MHz. These finding are in good agreement with previous reports on Cu^{2+} centers in distorted tetrahedral geometry[1] and help us to disambiguate the origin of the paramagnetic signal from the 3.4% Cu sample by assigning it to Cu^{2+} impurities possibly introduced either during the synthesis or present in the starting materials.

References:

1. Roger, M.; Biaso, F.; Castelle, C. J.; Bauzan, M.; Chaspoul, F.; Lojou, E.; Sciara, G.; Caffarri, S.; Giudici-Orticoni, M.-T.; Ilbert, M., Spectroscopic Characterization of a Green Copper Site in a Single-Domain Cupredoxin. *PLOS ONE* **2014,** *9*, e98941.
